# Effect of the pringle maneuver on tumor recurrence of hepatocellular carcinoma after curative resection (EPTRH): a randomized, prospective, controlled multicenter trial

**DOI:** 10.1186/1471-2407-12-340

**Published:** 2012-08-03

**Authors:** Feng Xiaobin, Zheng Shuguo, Zhou Jian, Qiu Yudong, Liang Lijian, Ma Kuansheng, Li Xiaowu, Xia Feng, Yi Dong, Wang Shuguang, Bie Ping, Dong Jiahong

**Affiliations:** 1 Institute of hepatobiliary surgery, Southwest Hospital, Third Military Medical University, Chongqing, P. R. China; 2 Institute of hepatobiliary surgery, Chinese PLA General Hospital, Beijing, P. R. China; 3 Institute of liver cancer, Zhongshan Hospital of Fudan University, Shanghai, P. R. China; 4 Department of hepatobiliary surgery, Nanjing Drum Tower Hospital, Nanjing, P. R. China; 5 Department of hepatobiliary surgery, the First Affiliated Hospital, Sun Yet-Sen University, Guangzhou, P. R. China; 6 Department of health statistics, Third Military Medical University, Chongqing, 400038, P. R. China

**Keywords:** Hepatocellular carcinoma, Ischemia/reperfusion, Hepatectomy, Pringle maneuver

## Abstract

**Background:**

Hepatic resection is currently still the best choice of therapeutic strategies for liver cancer, but the long-term survival rate after surgery is unsatisfactory. Most patients develop intra- and/or extrahepatic recurrence. The reasons for this high recurrence rate are not entirely clear. Recent studies have indicated that ischemia-reperfusion injury to the liver may be a significant factor promoting tumor recurrence and metastasis in animal models. If this is also true in humans, the effects of the Pringle maneuver, which has been widely used in hepatectomy for the past century, should be examined. To date, there are no reported data or randomized controlled studies examining the relationship between use of the Pringle maneuver and local tumor recurrence. We hypothesize that the long-term prognosis of patients with liver cancer could be worsened by use of the Pringle maneuver due to an increase in the rate of tumor recurrence in the liver remnant. We designed a multicenter, prospective, randomized surgical trial to test this hypothesis.

**Methods:**

At least 498 eligible patients from five participating centers will be enrolled and randomized into either the Pringle group or the non-Pringle group in a ratio of 1:1 using a permuted-blocks randomization protocol. After the completion of surgical intervention, patients will be included in a 3-year follow-up program.

**Discussion:**

This multicenter surgical trial will examine whether the Pringle maneuver has a negative effect on the long-term outcome of hepatocellular carcinoma patients. The trial will also provide information about prognostic differences, safety, advantages and disadvantages between Pringle and non-Pringle surgical procedures. Ultimately, the results will increase the available information about the effects of ischemia-reperfusion injury on tumor recurrence, which will be of immense benefit to general surgery.

**Trial registration:**

http://www.clinicaltrials.gov NCT00725335

## Background

Hepatocellular carcinoma (HCC) is one of the most common cancers worldwide, and has been ranked the second leading cancer killer in China since the 1990s [1]. Chronic hepatitis B virus (HBV) infection is the most common etiology in China [2].

Curative resection remains the best choice of HCC therapeutic strategies to date, but local tumor recurrence and remote metastasis unfortunately occur in many patients who have undergone surgery. Although there have been great advances in the diagnosis and treatment of HCC, the long-term prognosis is still unsatisfactory due to a high incidence of tumor recurrence, ranging from 50% to 60% [3-6]. The reason for this high recurrence rate is not entirely clear. Locoregional tumor recurrence with concomitant hepatic decompensation is the main cause of death. It is suggested that further strategies may be needed for the prevention and treatment of early and late recurrence [7].

The Pringle maneuver is a classical surgical technique widely used during hepatectomy since its advent in 1908[8]. During hepatic resection, severe bleeding represents a major life-threatening risk [9,10]. The Pringle maneuver (continuous or intermittent clamping of the hepatic artery and portal vein) is routinely used to reduce intraoperative bleeding [11,12]. It seems inevitable that maneuver causes ischemia-reperfusion (I/R) injury, resulting in complex metabolic [13,14], immunological [15], and microvascular [16-18] changes, which together might contribute to hepatocellular damage and dysfunction[13,19]. Over the past century, the effects of the Pringle maneuver have been widely discussed [8,20-32]. From the reported data, we know that the technique can significantly reduce blood loss during hepatectomy, meanwhile damaging the liver remnant through the I/R injury. Interestingly, the effects of the Pringle maneuver on the prognosis of oncology patients and on the behavior of tumor cells have not been specifically discussed. It remains unknown whether tumor recurrence and metastasis can be influenced by this surgical stress.

However, this possibility has been raised by the results of recent animal studies [33,34]. It has been shown that surgical stress, such as I/R injury, might cause delayed damage to the residual liver (“soil”), and may also affect the behavior of the tumor cells (“seeds”) by activating cell invasion and migration signal pathways, thus accelerating tumor recurrence. I/R injury tends to disrupt normal liver tissues and create an environment that may promote tumor recurrence. Hepatic resection will also induce tumor cells to become more aggressive by promoting the production of cytokines by nonparenchymal liver cells [35]. From the results of these studies, it can easily be concluded that I/R injury to the liver remnant may be a significant factor promoting tumor recurrence and metastasis in experimental animal models. Animal models may introduce potentially important concepts related to the mechanisms involved in tumor recurrence associated with surgical stress, specifically hepatic I/R injury. Furthermore, the promotion of liver metastasis by hepatic I/R injury during liver resection has been reported in colon cancer patients [36,37], and minimization of I/R injury can attenuate metastasis of colorectal cancer to the liver [38-40]. Liver transplant recipients with HCC who receive living-donor grafts experience a higher recurrence rate [41,42] due to the more severe acute-phase injury to the liver graft.

For primary liver cancer patient undergoing hepatectomy, the effects of I/R injury on tumor recurrence need to be determined. Because use of the Pringle maneuver during hepatic resection will lead to I/R injury to the liver remnant, we can rationally deduce that it may harm liver function, make the tumor cells more aggressive, and increase the likelihood of recurrence. If this is found to be true in humans, routine use of the Pringle maneuver should be re-evaluated. It is unfortunate that there are currently no reported data or randomized controlled studies examining this issue.

### Our hypothesis

We hypothesize that the high incidence of tumor recurrence and the poor long-term prognosis of patients with HCC might be partly due to the I/R injury resulting from use of the Pringle maneuver during hepatectomy. If possible, this procedure should be avoided or revised when performing a hepatectomy on cancer patients [43]. In a clinical setting, many factors such as blood loss, blood transfusion, liver function, and HBV load affect the prognosis of cancer patients. These factors may each have different, and sometimes even opposing, effects on laboratory test results. However, evaluation of the effects of the Pringle maneuver remains an important and urgent issue in liver surgery, which may lead to a significant change to our current knowledge. We therefore designed a multicenter, prospective, randomized trial to test our hypothesis.

## Methods

### Aims of the study

This trial aims to assess the long-term effects of the Pringle maneuver on the rate of tumor recurrence after curative resection of HCC. The effects on disease-free survival, overall survival, operative morbidity and mortality, duration of operation, blood loss, transfusion requirements, hospital stay, intensive care unit stay, and blood test results with prognostic relevance, will be examined. The trial will test whether hepatic resection without the Pringle maneuver reduces recurrence rate and improves disease-free survival rate.

### Trial population

Our study group includes researchers in five leading hospitals in China (Southwest Hospital, Chinese PLA General Hospital, Zhongshan Hospital, Nanjing Drum Tower Hospital, and the First Affiliated Hospital of SunYet-Sen University) located in the southwestern, northern, eastern, middle, and southern parts of China, respectively. All eligible patients from these five participating hospitals with a clinical diagnosis of HCC with infection will be enrolled. The study will include patients’ aged 18 years or older undergoing potentially curative (R0) resection, if preoperative imaging indicates that R0 resection can be undertaken both with and without the Pringle maneuver. Patients with extrahepatic disease, severe liver cirrhosis, or tumor-positive lymph nodes in the abdomen or hepatoduodenal ligament will be excluded. We anticipate that 50 eligible cases per month will be enrolled in the trial. A detailed list of all eligibility criteria follows.

### Eligibility criteria

 1. Aged from 18 to 65 years, no gender restriction.

 2. Clinical diagnosis of resectable HCC.

 3. Liver function tests showing Child-Pugh grade A and clearance of indocyanine green at 15 minutes (ICG-R15) less than 15%.

 4. Tumor nodes in the liver which can be radically excised.

 5. No preoperative anti-cancer therapy.

 6. Written informed consent from the patient or legal guardian prior to enrollment in the study.

### Exclusion criteria

 1. Pregnancy.

 2. Extrahepatic tumor or lymph node metastasis.

 3. 3.Tumor invasion or thrombosis in the portal vein, hepatic vein, or inferior vena cava.

 4. Surgical marginal positive.

### Study design

This study is funded by the National Major Science and Technology Project of China on the prevention and treatment of infectious diseases, for human immunodeficiency virus and HBV [2008ZX10002-026]. This clinical trial is a registered [NCT00725335], prospective, intraoperatively randomized multicenter trial of patients undergoing curative resection for HCC. The patients will be stratified by center and allocated to groups by a permuted-blocks randomization protocol. The primary objective of this study is to compare the disease free-survival rates at 1, 2, and 3 years postoperatively between the two groups. We hypothesize that the long-term prognosis of patients who have undergone curative resection will be worse in the control group (Pringle maneuver) than in the experimental group (non-Pringle maneuver).

**Figure 1:**
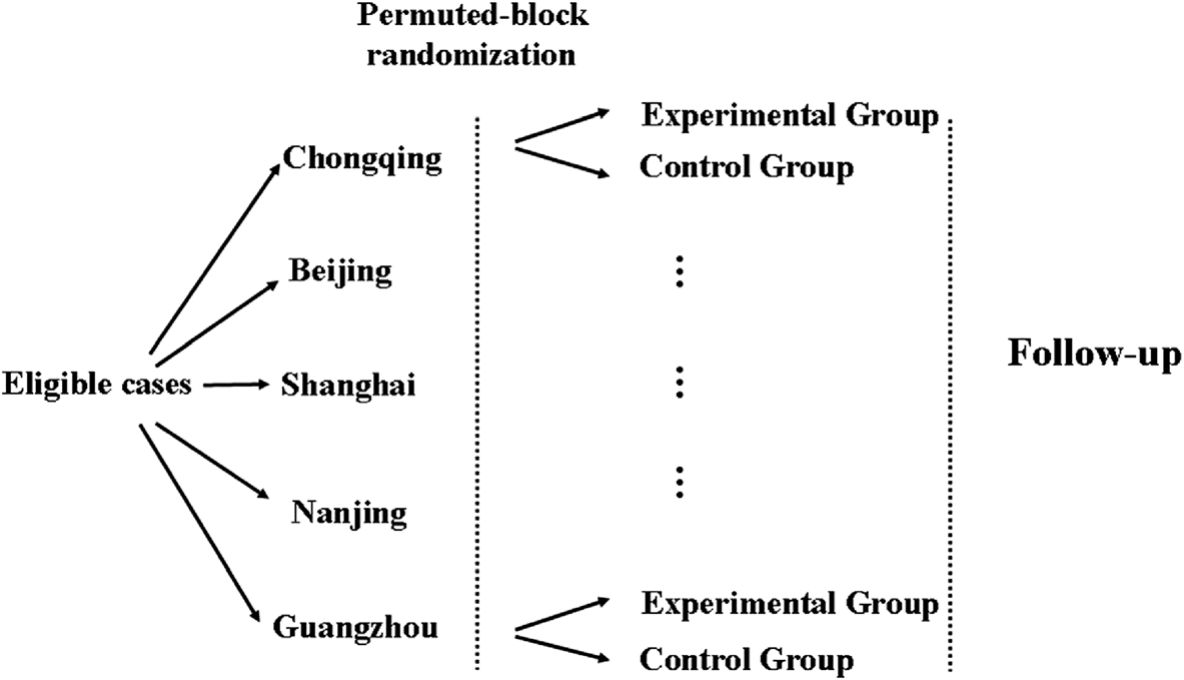
**Detailed flow chart of the clinical trial.** Eligible patients from five centers will be enrolled, and randomly divided into an experimental group and a control group by permuted-blocks randomization. Each patient will be followed up for 3 years after their primary operation.

The detailed study design is shown in Figure1.

### Surgical interventions

All surgical interventions in this trial are described in the study protocol.

### Incision lines and decision-making

Specific teams of surgeons have been designated in each center participating in this trial, and have completed the operative and postoperative management training for following the protocol. The abdominal incision can be decided according to the surgeon’s preference. A complete exploration of the abdomen and an intraoperative ultrasound evaluation of the whole liver will be performed to decide whether curative resection is feasible. If necessary, frozen section biopsies will be taken to evaluate suspicious lesions or lymph nodes. If both intervention procedures seem possible to the surgeon, randomization will be performed at this time point.

### Experimental group (group a, non-Pringle group)

The liver will be mobilized from the retroperitoneum and the peripheral ligament. Anatomical liver resection is preferable. If anatomical resection is not possible, a surgical margin of more than 2 cm should be achieved, except if the location of tumor nodes makes this impossible (e.g., close to the inferior vena cava or portal vein).The resection method and surgical margin will be recorded for analysis. The liver parenchyma can be resected according to the surgeon’s preference and local standards (CUSA, Tissue-link.). The Pringle maneuver will not be used, and hepatic inflow and outflow will be maintained. For safety, the surgeon may use the Pringle maneuver if major bleeding occurs.

### Control group (group B, Pringle maneuver group)

After mobilization, the Pringle maneuver will be performed. The same approach will be used for hepatic parenchymal transection as in the experimental group. If necessary, the outflow of the hepatic vein can be controlled, and this factor will be analyzed. For safety, the surgeon may change the surgical technique at any time during the operation.

### Pringle maneuver

The portal triad clamping is set to 15 min hepatic inflow occlusion followed by 5 min of reperfusion, repeated as needed. The last occlusion should be 15 min even if the transection has been completed. The total ischemia and reperfusion times and the number of occlusion cycles will be recorded for analysis. Any deviation in the standard operating procedures will be regarded as a protocol violation.

### Surgical team

The surgical teams consist of senior surgeons who all have standard qualifications and have independently performed standard anatomical hepatectomy in more than 100 patients. The results of randomization will be made known to the operating surgeon only after the disease has been deemed suitable for curative resection. All surgical procedures and anesthesia will be performed by the specifically trained teams of experienced hepatobiliary surgeons and anesthesiologists, ensuring standardized execution of the study protocol. Patients will be randomly assigned to eligible surgeons to minimize the effects of variations between surgeons on operative outcomes.

### Preoperative examination

Preoperative examination of patients will include blood biochemistry, alpha-fetoprotein assay, chest x-ray, percutaneous ultrasonography, computed tomography (CT) scan, and hepatic angiography in selected patients. Liver function will be assessed by Child-Pugh grading and the indocyanine green clearance test.

### Acquisition of samples

Blood samples: Two blood samples will be obtained from each patient after induction of general anesthesia through a central venous catheter, which is routinely placed just before surgery. EDTA (not heparin) will be used as an anticoagulant for blood samples. Follow-up blood samples will be collected every 6 months postoperatively.

Tissue samples: Paracarcinomatous tissue and cancer tissue will be harvested from the resected liver specimen at the time of resection. Paracarcinomatous tissue should be harvested 1 cm from the tumor margin. All tumors will be pathologically confirmed to be HCC.

Each tissue sample will be divided into two parts, one of which will be placed in liquid nitrogen and the other in 10% formalin. Serial 4-μm sections from each specimen will be stained with hematoxylin and eosin to determine clinicopathological features such as venous invasion, capsule formation, Edmondson’s grade, and cirrhotic nodules.

### Sample size calculation and data analysis plan

The sample size of this trial was calculated using data from our previous experience and from the published literature. There are currently no data from randomized controlled trials showing the recurrence rate of HCC after hepatectomy without the Pringle maneuver. Most studies used the Pringle maneuver. This trial plans to have equal numbers of control and experimental subjects, and a 36-month follow-up period. The median survival time after the treatment that the control group will receive has been reported to be 26 months [24,44-50]. We anticipate a10-month difference in the median recurrence-free survival time between the experimental and control groups. If median survival times in the control and experimental groups are 26 months and 36 months, respectively, we will need to include 249 experimental subjects and 249 control subjects to be able to reject the null hypothesis that the experimental and control survival curves are equal with a probability (power) of 0.800. The Type I error probability associated with this test of the null hypothesis is 0.05.

Data for primary and secondary outcome measures will be analyzed using the SPSS statistical software package (SPSS UK Ltd., Woking, U.K.). Analysis will include standard descriptive statistics, Student’s t tests, correlation and regression, and two-way (group x time) repeated measures ANOVA to examine differences between the groups over time. Survival analysis will be performed. Statistical significance will be set at p < 0.05and all tests will be two-tailed. Subgroup stratified analysis will be performed according to tumor size, tumor encapsulation, Edmondson’s grade, HBV load, ischemic time, blood loss, and transfusion. The intention-to-treat analysis will be used for the patients whose portal triad clamping was changed due to safety reasons.

### Randomization, stratification, and blinding

This is a randomized multicenter study. After giving informed consent and being enrolled in the study, patients will be randomized into different groups in the operating room after surgical exploration. The permuted-blocks randomization protocol will comprise five separate randomization lists, one for each participating hospital, and will be centrally managed by the Clinical Trial Center of the lead hospital, the Third Military Medical University. When an eligible case is enrolled, the Clinical Trial Center will be called and a randomization number will be assigned according to the designated list. Because there are two groups in this trial, the block sizes will be 4, 6, and 8 and will be randomly assigned. Patients will be stratified by site and randomized in a ratio of 1:1 into the two groups. Patients and outcome assessors will be blinded to achieve a minimum bias.

### Endpoints

The primary trial endpoint will be tumor recurrence diagnosed by enhanced CT scan or serum alpha-fetoprotein level. Secondary objectives are to examine overall survival, blood loss, duration of operation, requirement for blood transfusion, length of hospital stay, and morbidity rate. The formal end of the study will be at the end of the 3-year follow-up period of the last patient to be enrolled. All these parameters will be recorded prospectively as part of the study protocol. Final evaluation of the primary and secondary endpoints of the study will be performed 1 year after enrollment of the last patient.

### Clinical evaluation

The clinical evaluation of patients will consist of three stages: preoperative, intraoperative, and postoperative. During the preoperative stage, the surgeon will evaluate whether curative resection of the tumor can be performed with and without the Pringle maneuver. For safety reasons, the operating surgeon can change the portal triad clamping status at any time during the operation. If the portal triad clamping is changed, the patient will still be included in the intention-to-treat analysis. If a pathological diagnosis of non-primary HCC is made postoperatively by two different professionals who were blinded to the treatment, the patient will be excluded.

### Safety aspects and adverse advents

Both arms of this trial follow well-established procedures, which are widely used in many surgical centers allover the world. No specific side effects are expected other than the known complications of hepatectomy. The operating surgeon can change the portal triad clamping protocol at any time during the operation if necessary to ensure safety. All adverse events during the hospital stay and follow-up will be recorded for correlation analysis.

### Ethics and informed consent

The final version of the study protocol was approved by the ethics committee of Southwest Hospital, Third Military Medical University. This protocol follows all requirements of the recent German version of the Declaration of Helsinki (Somerset West Version, 1996) and is in accordance with the principles of Good Clinical Practice guidelines. The trial has been initiated and will be carried out following all local legal and regulatory requirements. The medical secrecy act will also be followed. Prior to enrollment in the study, written informed consent will be obtained from each patient in oral and written form. Any measures specifically required only for the clinical trial will not be undertaken until valid consent has been obtained. Extensive information about the intent of the study, the interventions in each group, the potential associated risks, and potential alternative therapies will be fully discussed with each patient. Patients will be also informed that participation is voluntary and can be withdrawn at any time without prejudicing their subsequent care. Patients will be informed of the strict confidentiality of their personal data collected for this trial, and that their medical records may be reviewed for trial purposes by authorized individuals.

### Follow-up

Patients who have completed the interventions will be included in the standard follow-up program. Follow-up visits will be at 1, 3, 6, 9, and 12 months and then every 6 months until 3 years after their operation. Each visit will include physical examination, tumor marker tests, liver function tests, chest x-ray, and ultrasound examination. In addition, enhanced CT scans of thorax and abdomen will be performed at 3, 6, 12, 18, 24, 32, and36 months postoperatively. Additional investigations including 18 F-fluorodexyglucose positron emission tomography scan, magnetic resonance imaging, or digital subtraction angiography will be scheduled as required to investigate possible tumor recurrence. At each visit, a follow-up evaluation form will be completed. If tumor recurs, the patient will be hospitalized and treated according to the clinical situation.

### Data management and monitoring

The Institute of Hepatobiliary Surgery of Southwest Hospital is responsible for the coordination of this trial. The clinical and laboratory data of all patients will be centrally collected and entered in a password-protected database at the Clinical Trial Center of Southwest Hospital, Third Military Medical University. All samples and clinical data will be tracked using a unique research tracking number. The link between research tracking numbers and patient identifiers will be kept in a limited-access database on a computer. Trial data quality reports will be generated routinely to evaluate missing data and inconsistencies. Accrual rates and follow-up will be monitored periodically throughout the study period. If a potential problem is identified, it will be brought to the attention of the investigator for discussion and treatment. All operation records will be reviewed to ensure that the study protocol was followed.

Monitoring of data and patient safety will be performed according to good clinical practice GCP guidelines by an independent Data and Safety Monitoring Board (DSMB) established by the Chinese PLA General Hospital. The DSMB will meet approximately twice a year to monitor safety and to advise the centers about study progress. In addition, the Clinical Trial Center will provide data to the DSMB Chair at regular intervals, and at his or her request, to ensure early identification of any major adverse outcomes of treatment. The DSMB will monitor adverse effects and respond to variations in the data, and is responsible for recommending whether the study should continue, whether the protocol should be modified, or whether there should be early termination.

### Duration of the trial, and current trial status

The trial is planned to last a total of 5 years, consisting of: protocol development (6 months), training (3 months), main recruitment (9 months), follow-up (36 months), and analysis (6 months). All the team members have completed their training. The expected end date of this trial will be July, 2013.

## Discussion

Even though short-term outcomes of the Pringle maneuver have been investigated, its effects on tumor recurrence and metastasis have not been discussed. This trial will focus on the effects of the I/R injury caused by the Pringle maneuver on the long-term prognosis of primary HCC patients with HBV infection.

Previous experimental studies have demonstrated that tumor recurrence could be increased by I/R injury to the liver remnant, and that the tumor cell might become more aggressive [33,35]. If this occurs in the clinical setting, use of the Pringle maneuver must be re-evaluated, because it has been widely performed in the past to avoid massive blood loss during hepatic resection. The Pringle maneuver may cause additional I/R injury besides that caused by the hepatic resection itself, which will affect tumor cells and the liver remnant.

With advances in surgical techniques and perioperative management, portal triad clamping is not necessary for every hepatectomy, and it may be important to further discuss its use. We hypothesize that the prognosis of patients with primary HCC might be worsened by use of the Pringle maneuver during hepatectomy. If this is true, this routine surgical technique should be revised or even avoided during hepatectomy, especially in HCC patients. We all know that it can take time for theories based on animal experiments to have an effect on clinical practice. In some cases, the final outcome may be affected by multiple clinical factors. For this trial, reported data indicate that these factors mainly include the hepatitis virus DNA load, surgical margins, microvascular thrombosis, tumor size, number of tumor nodules, microvascular invasion, tumor capsule formation, blood loss, and blood transfusion. We take all these factors into account in our inclusion and exclusion criteria and subgroup analysis. Whatever the results of this randomized controlled trial, we will gain important information about the long-term effects of the Pringle maneuver.

## Competing interests

All the authors declare that they have no competing interests.

## Pre-publication history

The pre-publication history for this paper can be accessed here:

http://www.biomedcentral.com/1471-2407/12/340/prepub
